# Promising Probiotic Properties of the Yeasts Isolated from *Rabilé*, a Traditionally Fermented Beer Produced in Burkina Faso

**DOI:** 10.3390/microorganisms11030802

**Published:** 2023-03-21

**Authors:** Iliassou Mogmenga, Marius Kounbèsiounè Somda, Cheik Amadou Tidiane Ouattara, Ibrahim Keita, Yérobessor Dabiré, Camelia Filofteia Diguță, Radu Cristian Toma, Lewis I. Ezeogu, Jerry O. Ugwuanyi, Aboubakar S. Ouattara, Florentina Matei

**Affiliations:** 1 Laboratoire de Microbiologie et de Biotechnologies Microbiennes, Université Joseph KI-ZERBO, Ouagadougou 03 BP 7021, Burkina Faso; 2 Department of Microbiology, Faculty of Biological Sciences, University of Nigeria, Nsukka 410001, Enugu State, Nigeria; 3 Centre Universitaire de Banfora, Université Nazi BONI, Bobo-Dioulasso 01 BP 1091, Burkina Faso; 4 Laboratoire de Biochimie, Biotechnologie, Technologie Alimentaire et Nutrition (LABIOTAN), Département de Biochimie Microbiologie, Université Joseph KI-ZERBO, Ouagadougou 03 BP 7021, Burkina Faso; 5 Faculty of Biotechnologies, University of Agronomic Sciences and Veterinary Medicine of Bucharest, 011464 Bucharest, Romania

**Keywords:** yeasts, *Rabilé*, Burkina Faso, *Saccharomyces*, non-*Saccharomyces*, probiotic properties

## Abstract

In recent years, research on yeasts as probiotics has gained more and more interest, which will allow the development of “new” products in the probiotics market. In this context, seventeen yeast strains isolated from *Rabilé*, a traditional beer produced in Burkina Faso, were assessed for their probiotic attributes. The yeast identification was performed by molecular methods, including PCR-RFLP and 5.8S-ITS region sequencing. *Saccharomyces cerevisiae* (14 strains) was the predominantly identified species, followed by *Pichia kudriavzevii* (2 strains) and *Rhodotorula mucilaginosa* (1 strain). Except for *R. mucilaginosa*, all yeast strains grew well at human temperature. The yeast strains showed high resistance when they were exposed to simulated gastrointestinal conditions. Auto-aggregation ability was between 70.20 ± 10.53% and 91.82 ± 1.96%, while co-aggregation with *E. coli* ranged from 24.92 ± 3.96% to 80.68 ± 9.53% and with *S. enterica serovar* Typhimurium from 40.89 ± 8.18% to 74.06 ± 7.94%. Furthermore, the hydrophobicity of isolated strains toward n-hexane was in the range from 43.17 ± 5.07% to 70.73 ± 2.42%. All yeast strains displayed high antioxidant capabilities, and the strains did not show hemolysis halos, such that they can be considered safe. Additionally, *S. cerevisiae* strains strongly inhibited the growth of foodborne pathogens. This is the first preliminary study to identify and characterize the yeast strains isolated from *Rabilé* with interesting probiotic properties.

## 1. Introduction

The current sustainability of food systems must be rethought in order to reduce hunger and prevent food and disease vulnerabilities in some parts of the world, mostly in developing countries [1,2,3]. For this reason, local fermented foods have been put back in the spotlight in Africa [3,4,5,6,7,8]. Several traditional fermented foods in Africa are obtained by the mediation of microorganisms (mainly lactic acid bacteria (LAB) and yeasts), such as *Rabilé* and *Ben-saalga* (Burkina Faso); Mawè (Benin); Gari, Fufu, and Kunu-zaki (Nigeria); kule naoto and Amabere amaruranu (Kenya); and Amasi and Mahewu (South Africa) (reviewed by Obafemi et al. [6]). According to FAO/WHO [9] and updates by Hill et al. [10], probiotics are defined as live microorganisms that confer beneficial effects on the host when administered in the proper amounts. Although several LAB species belonging to *Lactobacillus* and *Bifidobacterium* genera are the most studied and commercialized probiotics [11,12,13,14,15,16], yeast biotechnology is well-known and is used in the manufacturing of fermented foods and in starter/co-starter cultures in the development of new functional foods with high-value nutraceuticals [17,18,19,20,21,22,23]. In the last few years, research on yeasts as potential probiotics with valuable properties, which had previously been relatively neglected, has intensified to discover new “wild” yeast strains isolated from traditional fermented foods; such approaches may revolutionize the probiotics market, which has been dominated mainly by lactic acid bacteria [24,25,26,27,28,29,30]. *Saccharomyces cerevisiae* var. *boulardii* has been approved for commercial use as a probiotic yeast [31,32]. Additionally, *S. cerevisiae*, the best-known species, has been extensively studied for its valuable probiotic characteristics [33,34,35]. Currently, non-*Saccharomyces* species have been studied for their potential probiotic attributes [33,34,35,36,37,38,39]. Ogunremi et al. [36] have isolated new yeast strains from some traditional cereal-based fermented products from Nigeria, such as *Candida tropicalis*, *Issatchenkia orientalis*, *Pichia kudriavzevii*, and *Pichia kluyveri*, and characterized them as starter cultures with multifunctional potentials to produce cereal-based probiotic products. The probiotic properties of yeasts isolated from whole-grain millet sourdoughs have been evaluated [39]. According to the reviewed articles, the exploration of the probiotic potential of yeasts is based on the usual in vitro testing of the main functional characteristics, namely, species identification, safety requirements, the ability to survive the transition through the gastrointestinal tract (body temperature, stomach pH, various digestive enzymes, and bile salts), the ability to adhere to cell surfaces (hydrophobicity and self- and co-aggregation capacities), and antimicrobial activity [40,41,42,43]. Different *Saccharomyces* and non-*Saccharomyces* strains other than the commercially available *Saccharomyces boulardii* have been demonstrated to have valuable probiotic properties that could improve both human and animal health (modulating metabolism and immunity and antimicrobial activity), enhance livestock feed digestion and growth performance, and obtain functional foods/feeds, in addition to other novel applications [21,24,25,26,27,28,29,34,44,45,46].

LAB and yeasts have been reported as the main microorganisms isolated from traditional beers [6,47,48,49]. *Rabilé*, a traditional fermented beer from Burkina Faso, is also used as a condiment to supply protein in cereal-based foods [49]. To our knowledge, research on the probiotic properties of yeasts isolated from *Rabilé* has not been carried out.

Hence, the goal of this research was to investigate in vitro the functional and probiotic properties of yeast strains isolated from *Rabilé* as a basis for establishing the nutraceutical value of *Rabilé*.

## 2. Materials and Methods

### 2.1. Microorganisms and Growth Conditions

For this study, 17 yeast strains isolated from *Rabilé* beer [49] were selected to evaluate their probiotic properties. Strains were maintained by cultivation in yeast extract dextrose-peptone broth (YPD) (containing 2% *w/v* dextrose, 2% *w/v* peptone, and 1% *w/v* yeast extract) at 30 °C for 24 h and stored in the presence of 20% (*v*/*v*) glycerol at −20 °C.

*Escherichia coli* ATCC 8739, *Listeria monocytogenes* ATCC 7644, *Salmonella enterica* serovar Typhimurium ATCC 14028, *S. enteritidis* ATCC 13076, and *Staphylococcus aureus* ATCC 33592 (provided by the American Type Culture Collection (ATCC) (Manassas, VA, USA) were used as the reference pathogenic bacteria for the antimicrobial tests, while *E. coli* ATCC 8739 and *S. enterica* serovar Typhimurium ATCC 14028 were used in the co-aggregation assays. Pathogenic bacteria were maintained by cultivation in tryptic soy broth (TSB) or tryptic soy agar (TSA) (Scharlab, Barcelona, Spain) at 37 °C for 24 h.

### 2.2. Molecular Identification of Yeast Isolates

#### 2.2.1. Amplification of the 5.8S-ITS Region and RFLP Analysis

The DNA extraction was performed with fresh yeast cultures using the Quick-DNA™ Fungal/Bacterial Miniprep Kit (Zymo Research, Irvine, CA, USA), according to the supplier’s instructions, and the extracts were stored at −20 °C until use. The universal primers ITS1 (5’-TCCGTAGGTGAACCTGCGG-3’) and ITS4 (5’-TCCTCCGCTTATTGATATGC-3’) [50] were used to amplify the 5.8S-ITS region by PCR. All PCRs were carried out in 50 μL of solution containing 5 µL of 10X DreamTaq Green Buffer supplemented with MgCl_2_, 2.5 µL of each 10 µM primer, 1 µL of 10 mmol dNTP, 0.25 µL of 1U DreamTaq polymerase (Thermo Fisher Scientific, Baltics, UAB, Vilnius, Lithuania), and 10 ng·μL^−1^ fungal DNA. PCR amplifications were performed in a MultiGene PCR System (MyCycler thermal cycler, BIO-RAD, Hercules, USA) under the conditions described by Esteve-Zarzoso et al. [51]. Then, without further purification, each PCR product was digested separately with three restriction enzymes—*Hae*III, *Hinf*I, and *Hha*I (Thermo Fisher Scientific, Baltics, UAB, Vilnius, Lithuania)—at 37 °C for a minimum of 2 h, according to the supplier’s instructions. PCR products and their restriction fragments were separated on 2% (*w*/*v*) agarose gel, and their lengths were approximated by comparison with the known DNA size standards (GeneRuler 100 bp Plus DNA Ladder; Thermo Fisher Scientific, Baltics, UAB, Vilnius, Lithuania). The electrophoretic patterns were captured using the GelDoc-It Imaging System (Analytik Jena, Upland, CA, USA). The RFLP patterns were compared with restriction analyses performed by Esteve-Zarzoso et al. [51] and with those of the reference yeast species freely available in the Yeast-id database (http://www.yeast-id.com, accessed on 29 November 2021). The similarity of restriction patterns was analyzed using Pearson’s correlation coefficient r. The yeast strains were grouped using the unweighted pair group method with arithmetic averages (UPGMA) [52].

#### 2.2.2. Yeast Strain Identification by Sequencing

The 5.8-ITS sequences of some yeast isolates belonging to the different detected yeast groups were sequenced by the Cellular and Molecular Immunological Application (CEMIA, Greece) to confirm the identifications at the species level by ITS-RFLP. The sequenced sequences were submitted using BLASTN tools (http://www.ncbi.nlm.nih.gov/BLAST/, accessed on 14 February 2022) for alignment with different sequences available in the NCBI database based on similarity percentages.

### 2.3. Growth Capacity at 37 °C

Quantities of 10 mL of YPD Broth in sterile tubes were inoculated with fresh yeast cultures (adjusted to OD600 at 0.3 ± 0.05). After incubation at 37 °C for 24 h, the growth rates of the yeast strains were recorded by measuring optical densities at 600 nm.

### 2.4. Survival of Simulated Gastrointestinal (GI) Digestion

#### 2.4.1. Tolerance of Pepsin Presence and Acidic pH

The pepsin and pH 2.5 tolerance tests of the yeast strains were performed according to Burns et al. [53]. Briefly, overnight yeast cultures grown in YPD broth were centrifuged (4000× *g*, 5 min). Cell pellets were washed twice with sterile phosphate buffer (PB) (pH 7.0) (0.5382% (*w*/*v*) NaH_2_PO_4_ and 1.6363% (*w*/*v*) Na_2_HPO_4_.7H_2_O) and resuspended in 10 mL of sterile PB with low pH (2.5) and containing pepsin (0.3%). Then, the samples thus prepared were incubated at 37 °C for 3 h in static conditions. Aliquots were taken at 0 h (initial) and after 3 h (final) and diluted in sterile saline 0.85% solution. Survival rates were assessed by the drop plate method on YPD Agar and were calculated using the following formula:

$$\mathrm{SR}\%=\frac{\mathrm{Log}\;\mathrm{CFU}/\mathrm{mL}\;\left(\mathrm{final}\right)}{\mathrm{Log}\;\mathrm{CFU}/\mathrm{mL}\;\left(\mathrm{initial}\right)}\times 100$$

#### 2.4.2. Tolerance of Bile Salts

Bile salt tolerance of the yeast strains was determined following the method reported by Pedersen et al. [54], with a few modifications. The YPD broth supplemented with bile salts (0.3%) (HiMedia Laboratories, Pvt. Ltd., Maharashtra, India) was inoculated with fresh yeast cultures and then incubated at 37 °C for 4 h. Aliquots were taken at 0 h (initial) and after 4 h (final) and diluted in a sterile saline 0.85% solution. Survival rates were assessed by the drop plate method on YPD Agar and were calculated using the following formula:

$$\mathrm{SR}\%=\frac{\mathrm{Log}\;\mathrm{CFU}/\mathrm{mL}\;\left(\mathrm{final}\right)}{\mathrm{Log}\;\mathrm{CFU}/\mathrm{mL}\;\left(\mathrm{initial}\right)}\times 100$$

### 2.5. Auto-Aggregation Ability

Auto-aggregation assays were performed according to Binetti et al. [55]. Briefly, the fresh yeast cells were collected by centrifugation (4000× *g*, for 10 min) and washed twice with 0.2 mol·L^−1^ PB solution (pH 7.2). The OD600 was adjusted to 1.0 ± 0.05. The cell pellets were resuspended in 2 mL sterile PB by vortexing for 30 s, then incubated at 37 °C for 24 h. Aliquots of these suspensions (1 mL) were carefully removed from the upper zone to measure OD at 600 nm. Auto-aggregation ability was calculated as:

$$\mathrm{Auto}-\mathrm{aggregation}\;\left(\%\right)=1-\frac{{\mathrm{DO}}_{0}}{{\mathrm{DO}}_{t}}\times 100$$

where OD_0_ and OD_t_ are the optical densities at 0 h and after 24 h, respectively.

### 2.6. Co-Aggregation Activity

The co-aggregation assays were assessed using the method described by Kos et al. [56], with some modifications. *E. coli* ATCC 8739 and *S. enterica* serovar Typhimurium ATCC 14028 were used as target bacteria. The cell suspensions were prepared as above. A quantity of 2 mL of bacterial suspension was mixed with an equal volume of yeast suspension by vortexing for 10 s and then incubated at 37 °C for 24 h. Unmixed yeast and bacterial suspensions were used as controls, under the same conditions. After incubation, the absorbances (ODs) of all suspensions were measured at 600 nm. The percentage of co-aggregation was expressed as:

$$\mathrm{Co}-\mathrm{aggregation}\;\left(\%\right)=\frac{({\mathrm{OD}}_{\mathrm{yeast}}+{\;\mathrm{OD}}_{\mathrm{pathogen}})/2)-\mathrm{OD}\left(\mathrm{yeast}+\mathrm{pathogen}\right)}{({\mathrm{OD}}_{\mathrm{yeast}}+{\;\mathrm{OD}}_{\mathrm{pathogen}})/2}\times 100$$

The percentages of auto-aggregation and co-aggregation were considered low (below 30%), intermediary (between 30% and 60%), or high (greater than 60%) [41].

### 2.7. Hydrophobicity

Cell surface hydrophobicity was determined according to the method of Alkalbani et al. [42]. Overnight yeast cultures were centrifuged at 4000× *g* for 10 min. Cell pellets were washed twice with 0.1 mol·L^−1^ PB solution (pH 7.0) and adjusted to the OD600 at 1.0 ± 0.05 (ODi). Quantities of 3 mL of cell suspensions were mixed well with 0.6 mL of n-hexane (VWR International, Rosny-sous-Bois, France) by vortexing (10 s). After incubation at 37 °C for 3 h, the aqueous phases were carefully recovered and OD600 nm values were measured (ODf). Cell surface hydrophobicity (H%) was determined using the following formula:

$$H\%=\frac{{\mathrm{OD}}_{i}-{\;\mathrm{OD}}_{f}}{{\mathrm{OD}}_{i}}\times 100$$

### 2.8. Hemolytic Activity

Hemolytic activity was conducted by spot-inoculating the yeast strains on Columbia Agar with Sheep Blood Plus plates (Oxoid, UK). After inoculation, the plates were incubated at 37 °C for 48 h according to the methodology used by Menezes et al. [57]. The occurrence of a clear area (denoted as β-hemolysis) or a green-hued zone surrounding the yeast colonies (denoted as α-hemolysis) was considered a positive result, which meant disqualification as a probiotic [9]. Only the yeast strains that showed neither hemolysis halos nor green-hued zones after growth on blood agar plates (denoted as γ-hemolysis) were considered safe and used for the next studies [9].

### 2.9. Antioxidant Activity

The antioxidant activities of the yeast strains were determined using the methodology described by Chen et al. [58], with slight modifications. Briefly, the yeast cell suspensions were prepared as above. Quantities of 500 μ L of cell suspension were mixed with 1 mL of 1,1 diphenyl-2-picrylhydrazyl (DPPH) solution (0.2 mM in methanol) and vigorously vortexed for 2 min. After incubation at room temperature in darkness for 30 min, the supernatants were recovered by centrifugation (10,000× *g*, 5 min), and their absorbances were measured at 517 nm. Deionized water was used in the control solution. The percentage of DPPH radical scavenging was calculated as:

$$\mathrm{Ac}\;\left(\%\right)=\frac{1-\mathrm{OD}\left(\mathrm{sample}\right)}{\mathrm{OD}\left(\mathrm{control}\right)}\times 100$$

### 2.10. Antibacterial Activity

The antibacterial activities of the yeast strains were assessed by employing the cross-streaking method described by Diguță et al. [33] against *E. coli* ATCC 8739, *L. monocytogenes* ATCC 7644, *S. enteritidis* ATCC 13076, *S. enterica* serovar, Typhimurium ATCC 14028, and *S. aureus* ATCC 33592. Fresh yeast cultures were first inoculated as 90 mm long streaks in the middle of Petri dishes on YPD agar and grown at 37 °C for 48 h. Subsequently, each indicator bacterium (overnight culture) was deposited by drawing a streak perpendicular to the previously seeded yeast strains, very close to but without touching them, and again incubated at 37 C for 24 h. The antibacterial activities of the yeast strains were recorded by measuring the bacterial growth inhibition sizes in millimeters (mm) using a ruler.

### 2.11. Statistical Analyses

All assays were repeated in triplicate, and the results were recorded as the means ± standard deviations (SDs). Analysis of variance (ANOVA) was used to compare the means of different variables, with the significance level at *p* = 0.05. The differences between the means of the tests were evaluated by the Fisher HDS test. Additionally, principal component analysis was performed to identify the correlations between the different variables and to select the yeasts with valuable probiotic attributes.

## 3. Results

### 3.1. Molecular Identification of Yeast Strains

A total of 17 yeast strains isolated from *Rabilé*, a traditional fermented beer, were investigated in this study. The morphological, cultural, and physiological characteristics of these yeast strains were determined, and the results were published elsewhere [49]. The yeast identifications at the species level were conducted by molecular methods, including PCR-RFLP and 5.8S-ITS region sequencing. According to the sizes of the PCR products, three groups were identified: group I (850 bp), group II (620 bp), and group III (510 bp) (Table 1). ITS-RFLP analysis confirmed the presence of three different groups (species). Fourteen yeast isolates displayed RFLP patterns corresponding to *Saccharomyces cerevisiae*, two isolates belonged to *Pichia kudriavzevii*, and one isolate belonged to *Rhodotorula mucilaginosa* (Table 1).

**Figure 1:**
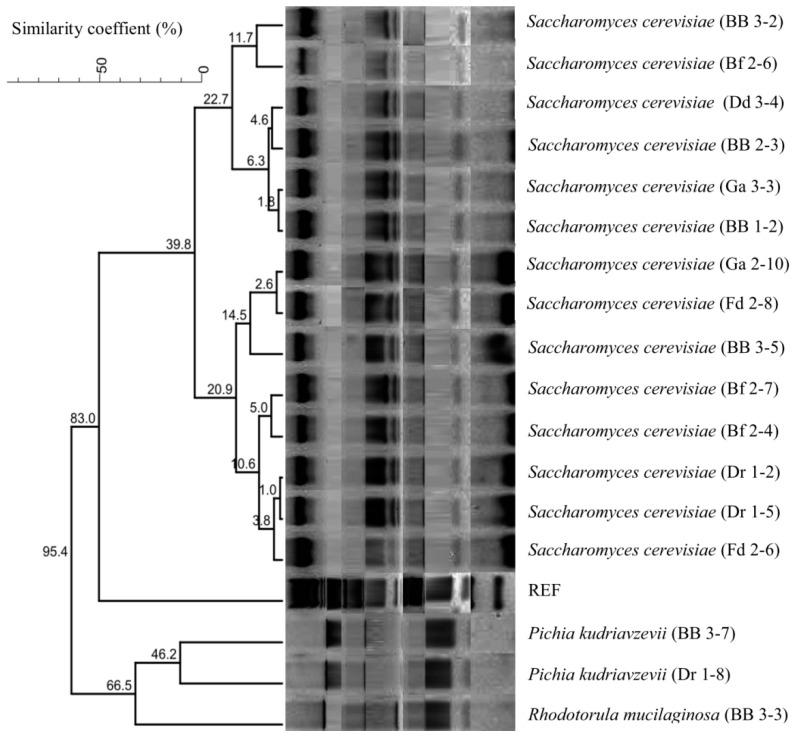
Dendrogram produced by comparing the RFLP-PCR profiles of yeast strains with the restriction enzymes *Hinf*I, *Hha*I, and *Hae*III with the PCR product profiles using Gel Compar Software, Applied Maths, Sint-Martens-Latem, Belgium, and clustering the data using the UPGMA method.

The dendrogram obtained by the comparison and clustering of RFLP patterns of yeast strains with the restriction enzymes *Hinf*I, *Hha*I, and *Hae*III and the amplicon profiles revealed the existence of the family relationships between the strains examined in this study (Figure 1).

The identifications at the species level obtained by ITS-RFLP analysis were confirmed by sequencing of the 5.8-ITS sequence of one yeast strain from each group and validated by high similarity percentages (from 99.35% to 100%) with respect to different ITS sequences available in the NCBI databases. In addition, the sequences stored in the NCBI database can be downloaded using the accession numbers OQ179952 (*S. cerevisiae* Ga 2-10), OQ179953 (*R. mucilaginosa* BB 3-3), and OQ179954 (*P. kudriavzevii* BB 3-7).

### 3.2. Tolerance at 37 °C (Body Temperature)

The yeast strains were able to grow at 37 °C (body temperature), with OD600 nm values ranging between 1.68 ± 0.11 (*P. kudriavzevii* Dr 1-8) and 4.27 ± 0.05 (*S. cerevisiae* BB 3-5) (Table 2). However, the *R. mucilaginosa* BB 3-3 strain did not grow very well at 37 °C, which means that this strain may not be suitable for human probiotics (Table 2).

### 3.3. Survival of Simulated Gastrointestinal Conditions

#### 3.3.1. Tolerance of Pepsin 0.3% and pH 2.5 at 37 °C

The effects of the simulated gastric conditions (0.3% pepsin and pH 2.5) on cell viability are presented in Table 2. The survival rates of the yeast strains varied from 86.01 ± 1.98% to 99.98 ± 0.00%. The *S. cerevisiae* strain (Bf 2-4) showed the lowest rate, while the *S. cerevisiae* strain (BB 1-2) showed the highest survival rate.

#### 3.3.2. Bile Salt Tolerance

All of the yeast isolates tested were able to tolerate 0.3% bile salts. The *S. cerevisiae* BB 2-3 strain showed the lowest percentage (95.41 ± 2.74%). The *P. kudriavzevii* strains (BB 3-7 and Dr 1-8) and the *S. cerevisiae* strains (Bf 2-4, Fd 2-8, and Ga 2-10) showed the highest survival percentages (100.00 ± 0.00%) (Table 2).

### 3.4. Auto-Aggregation, Co-Aggregation, and Hydrophobicity of Yeast Isolates

The results obtained for the auto-aggregation, co-aggregation, and hydrophobicity activities of the 17 yeasts strains are displayed in Table 3. The cell surface hydrophobicity and auto-aggregation capacities of probiotic candidates are considered important for the overall ability to adhere to a hydrocarbon solvent and epithelial cells and intestinal mucosa, these factors being relevant to the ability to colonize the GI tract. As shown in Table 3, the auto-aggregation rate varied between 70.20 ± 10.53% and 91.82 ± 1.96% (*p* < 0.05) after 24 h of incubation.

Furthermore, co-aggregation activities were evaluated to investigate the abilities of the yeast strains to prevent the colonization of the intestine by pathogens. In our study, the co-aggregation percentages of the yeast strains with *E. coli* ATCC 8739 ranged from 24.92 ± 3.96% to 80.68 ± 9.53% and with *S. enterica serovar* Typhimurium ATCC 14028 from 40.89 ± 8.18% to 74.06 ± 7.94%, with significant differences (*p* < 0.05) among the isolates (Table 3).

The hydrophobicities of the yeast strains toward n-hexane varied considerably (Table 3), with values in the range from 43.17 ± 5.07% to 70.73 ± 2.42% (*p* = 0.05). The highest hydrophobicity values were determined for the *S. cerevisiae* strain Fd 2-6 (70.73 ± 2.42%), *R. mucilaginosa* BB 3-3 (68.99 ± 5.37%), and *P. kudriavzevii* Dr 1-8 (68.84 ± 5.23%), respectively.

### 3.5. Hemolytic Activity

According to FAO/WHO [9], the safety aspects of any probiotic candidate should be considered, including specifications and lack of harmful activities. Probiotic organisms should be GRAS-compliant. In our study, all yeast strains demonstrated γ-hemolysis activity, which demonstrated their safety for use.

### 3.6. Antioxidant Activity

**Figure 2:**
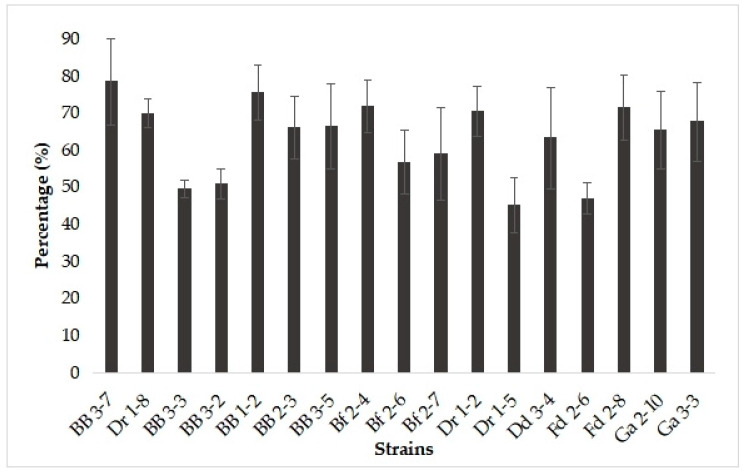
Antioxidant activities (%) of the yeast isolates evaluated by DPPH reduction.

DPPH free radical scavenging is one of the methods most often used to evaluate the antioxidant potential of probiotic candidates. The antioxidant activities of intact yeast cells were in the range of 45% to 78% (Figure 2). Among all 17 isolates studied, *P. kudriavzevii* BB 3-7 exhibited the highest percentage of DPPH reduction, and *S. cerevisiae* strains Dr 1-5 and Fd 2-6 exhibited the lowest values.

### 3.7. Antibacterial Activities of the Yeast Strains

The antibacterial activities of the yeast strains were assessed against foodborne bacteria, including Gram-positive bacteria (*L. monocytogenes* and *S*. *aureus*) and Gram-negative bacteria (*E. coli*, *S. enteritidis*, and *S. typhimurium*). As stated in Table 4, *S. cerevisiae* strains were the most effective in inhibiting the growth of all tested target pathogenic bacteria. Among *Saccharomyces* strains, the Fd 2-6, Dd 3-4, Ga 2-10, BB 3-2, and Bf 2-6 strains showed high antibacterial activity with large clear zones of inhibition ranging between 20.00 ± 0.00 mm and 30.00 ± 0.00 mm in size (Table 4). *P. kudriavzevii* strains (BB 3-7 and Dr 1-8) showed low antibacterial activity, and the *R. muciloginosa* strain (BB 3-3) showed no activity against pathogenic bacteria (Table 4).

### 3.8. Selection of Yeasts with the Highest Probiotic Potential

A PCA was carried out on 17 yeast isolates and the variables of probiotic properties were determined; this allowed the clustering of yeast strains according to their potential probiotic properties.

**Figure 3:**
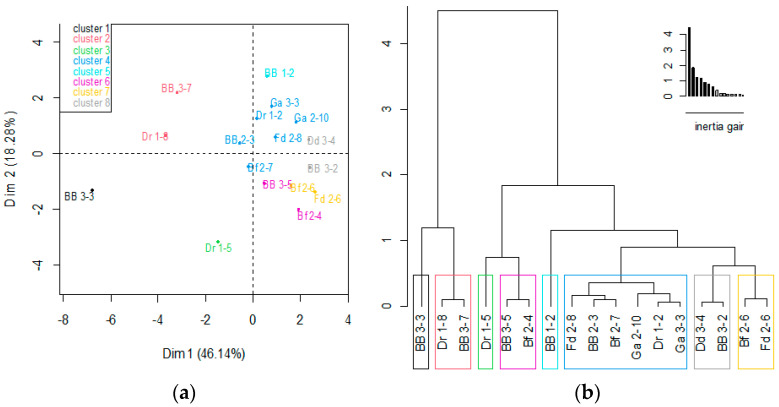
Ascending hierarchical classification of the individuals (**a**) and the hierarchical tree (**b**).

The classification of strains according to their probiotic properties is presented in Figure 3a. The hierarchical tree presented in Figure 3b shows eight clusters. Cluster 1 (BB 3-3 strain) was characterized by low values for the variable tests, including 0.3% bile salts, tolerance at 37 °C, and no antibacterial activity. Cluster 2 (Dr 1-8 and BB 3-7 strains) and cluster 3 (Dr 1-5) were characterized by low values of antibacterial activity and low tolerance at 37 °C. Next, cluster 4 (Ga 2-10, Dr 1-2, Ga 3-3 Bf 2-7, Bf 2-6, BB 2-3, and Fd 2-8 strains), cluster 5 (BB 1-2 strain), and cluster 6 (BB 3-5 and Bf 2-4 strains) were characterized by variables whose high values did not differ significantly from the means. Cluster 7 (Fd 2-6 strain) and cluster 8 (Dd 3-4 and BB 3-2 strains) were distinguished by high values for the variable tests.

## 4. Discussion

Yeasts have been intensively studied and exploited as starter cultures to produce fermented foods [2,3,4,5,6,7,18,19,20,21,22,23]. Recently, *Saccharomyces* and non-*Saccharomyces* strains isolated from African fermented foods have been studied and promoted as promising probiotics with health benefits [4,8,35,36,47,48,49,54,59]. In our study, the molecular identification of yeast strains isolated from *Rabilé* beer revealed the presence of three species—*S. cerevisiae*, *P. kudriavzevii*, and *R. mucilaginosa*—and the predominance of *S. cerevisiae*. *S. cerevisiae* has been reported as the predominant yeast isolated from fermented foods and beverages, being known to enhance nutritional and organoleptic properties, as well as probiotic properties [8,33,34,35,60,61]. Recently, *P. kudriavzevii* strains have been proposed as probiotic candidates and have been found to enhance fermented African foods [36,59].

Further, the yeast strains were investigated to see whether they qualified as probiotics based on several criteria mentioned in review papers [40,41,42,43]. To exert probiotic effects, yeast strains should tolerate low pH levels and bile salts and also be adaptable to corporeal temperature. All strains were subjected to acidic conditions (pH 2.5) and 0.3% pepsin at a temperature of 37 °C—the relevant conditions for the stomach. Our yeast strains were isolated from low-pH environments (*Rabilé*) [49] where they co-existed with LAB, which could explain their tolerance of acidic conditions. Diguță et al. [33] reported the abilities of *M. pulcherrima* OBT05, *S. cerevisiae* BB06, and *T. delbrueckii* MT07 to grow well in large pH ranges (1.5–7.5). Similar studies confirmed the tolerance of low pH of yeast strains isolated from different indigenous fermented foods [35,36,62]. Most natural or traditional fermented foods, including African traditional alcoholic beverages, such as palm wines, and partially fermented, grain-based gruels and beverages are produced by yeasts or LAB, as well as multi-strain mixtures [3,7,8,18,36].

In addition to low-pH tolerance, tolerance of bile salts is considered to be an essential criterion for the evaluation of potential probiotics to exert influence in the GI tract beyond the small intestine [42,53]. These bile salts have antimicrobial activities, and to reach the intestinal tract in a viable form any ingested microorganism has to resist them, otherwise it will be unable to withstand the presence of bile in the duodenum [40,42]. Our yeast strains showed high survival rates in artificial gastric juice conditions (between 86.01 ± 1.98% and 99.98 ± 0.00%) and stronger tolerances at the 0.3% bile salt concentration (between 95.41 ± 2.74% and 100.00 ± 0.00%), which are supported by previously reported results. In the study by Ogunremi et al. [36], *Issatchenkia orientalis*, *P. kluyveri*, and *P. kudriavzevii* strains showed tolerance of 2% bile salts. Additionally, Adisa et al. [39] reported that *Kluveromyces lactis* and *S. cerevisiae* tolerated up to 2% bile salt concentrations.

A prerequisite for the persistence of yeast probiotics in the GI tract is their capacity to adhere to the intestinal mucosa. In the current study, our yeast strains revealed high percentages of auto-aggregation (>70%) after 24 h, confirming the results reported in other research. Variable results were found by Fernandez-Pacheco et al. [62] after 30 min of incubation (3.85–64.43%), with the best percentages determined for *Hanseniaspora osmophila* and *Candida pararugosa*. Indeed, auto-aggregation increases the time yeast strains can spend in the intestine and allows them to have a positive impact on health. Menezes et al. [57] found variable results (66.0 ± 2.1% and 99.3 ± 0.6%), with the best auto-aggregation percentages shown by the *S. cerevisiae* strain CCMA 0716. In their study, Fernandez-Pacheco et al. [62] reported that percentages of auto-aggregation varied between 17% and 62% after 30 min of incubation, with *R. muciloginosa* 32 showing the highest value. Diguță et al. [33] reported the strongest auto-aggregation capacity of *S. cerevisiae* BB06 (92.08 ± 1.49%) after 24 h. According to these results, some researchers have reported that the auto-aggregation of yeasts seems to be a strain-dependent property [36,57]. The high percentages of auto-aggregation observed in yeasts may derive from the fact that yeast cells are relatively large and heavy and precipitate relatively quickly [36,40,41,42]. Trunk et al. [63] reported that the mechanism of bacterial auto-aggregation can involve simple surface electrostatic effects due to charges on bacterial surfaces. Furthermore, the co-aggregation abilities of yeasts can be considered as strategies to prevent the attachment and subsequent colonization of pathogens. Our yeast strains were able to co-aggregate with the tested bacterial pathogens, with the highest percentage obtained for *R. mucilaginosa* BB 3-3 with *E. coli* and for *P. kudriavzevi*i BB 3-7 with *S. enterica serovar* Typhimurium. Ogunremi et al. [36] reported a high co-aggregation ability of *P. kudriavzevii* OG32 with *E. coli* (71.57%). From the point of view of probiotic activity, the high hydrophobicity of cell surfaces could be the reason why some strains have distinct health benefits and slower elimination kinetics in the GI tract [42]. However, the varying degrees of adherence of our yeast strains to n-hexane were observed in a range between 43% and 71%. Binetti et al. [55] reported hydrophobicity values that ranged from 45.3% to 85.5% for yeasts isolated from autochthone cheese. Ogunremi et al. [36] evaluated the hydrophobicities of *I. orientalis*, *P. kluyveri*, and *P. kudriavzevii* strains isolated from cereal-based, traditional fermented food products of Nigeria with respect to toluene and octane and found good hydrophobic affinity to n-hexadecane (33.61–42.30%) in *P. kudriavzevii* strains. With xylene as a hydrocarbon, Fernandez-Pacheco et al. [62] found hydrophobicity rates that ranged between 2.6% and 34.6%, with the highest value obtained for *Meyerozyma caribbica* 35. In general, the capability of microorganisms to adhere to surfaces is a complex, multistep process that includes hydrophobic forces, electrostatic interactions, and interactions between the physical and chemical properties of the microbial surface and intestinal mucosa [29,42]. Cell surface hydrophobicity and auto-aggregation capability are the main parameters of probiotic candidates relevant to adherence to the intestinal epithelium of the host and the formation of biofilms [40,41,42].

The natural antioxidant capacities of yeast cells have been reported in several studies [29,33,58]. Chen et al. [58] reported a higher antioxidant capacity of intact yeast cells than cell extracts. One explanation would be the high content of (1/3)-β-D- glucan and other β-glucans found in the yeast cell wall and, additionally, antioxidant enzymes, such as catalase, superoxide dismutase, and glutathione peroxidase. According to their percentages of antioxidant activity, Gil-Rodríguez et al. [29] classified yeasts into five groups: very low (< 20%), low (20–30%), good (30–40%), very good (40–50%), and excellent (> 50%). Based on this grouping, the yeast isolates in the present study were classified into two levels: three isolates (BB 3-3, Fd 2-6, and Dr 1-5) showed very good activity, and fourteen (Ga 2-10, Ga 3-3, Bf 2-6, Dd 3-4, Fd 2-8, Dr 1-2, BB 3-2, BB 3-7, BB 3-5, BB 1-2, Bf 2-7, Bf 2-4, Dr 1-8, and BB 2-3) showed excellent activity.

From a biotechnological point of view, antimicrobial activity is an essential criterion, since yeast strains could be used in biological controls and food preservation and as promising probiotic candidates with health benefits. Our research revealed that only *S.cerevisiae* strains showed high antibacterial effects against bacterial pathogens, such as *E. coli*, *L. monocytogenes*, *S*. *aureus*, *S. enteritidis*, and *S. typhimurium.* In another work, Diguță et al. [33] demonstrated the high antibacterial activity of *S. cerevisiae* BB06 against nine foodborne pathogenic bacteria, namely, *Bacillus cereus*, *Enterococcus faecalis*, *E. coli*, *L.monocytogenes*, *L. ivanovii*, *Proteus vulgaris*, *Pseudomonas aeruginosa*, and *S. aureus*. The antimicrobial activities of yeast against pathogens could be due to the competition for nutrients and, simultaneously, the production of organic acids, hydrogen peroxide, and diacetyl. In another study, Adisa et al. [39] reported the antimicrobial activities of *S. cerevisiae* against *Klebsiella* spp. and of *K. lactis* against *Pseudomonas* spp. and *Staphylococcus* spp. Fernandez-Pacheco et al. [62] also reported, among 20 yeast isolates, 1 yeast (*Diutina rugosa* 12) that presented antimicrobial activity against *Dekkera bruxellensis* and *Zygosaccharomyces* spp.

According to the PCA analysis, *S. cerevisiae* strains (especially Fd 2-6, Dd 3-4, and BB 3-2 strains) were selected as having numerous valuable probiotic properties and could be potential candidates for establishing the high nutraceutical value of *Rabilé* beer.

## 5. Conclusions

In this study, 17 yeast strains isolated from traditional *Rabilé* beer produced in Burkina Faso were identified by molecular methods as *S. cerevisiae* (14 strains), *P. kudriavzevii* (2 strains), and *R. mucilaginosa* (1 strain). Selecting a yeast strain with all the desired probiotic attributes tested proved to be difficult. However, the strains evaluated in this study have many of the essential and critical probiotic characteristics that recommend them for probiotic use. These strains were able to grow at human body temperature (except *R. mucilaginosa*) and survive in the gastrointestinal tract. Meanwhile, *S. cerevisiae* strains showed strong antibacterial activities against the pathogens that were used. Additionally, these strains exhibited high antioxidant properties. Furthermore, they showed high percentages of hydrophobicity and strong auto-aggregation abilities, as well as various degrees of co-aggregation with *E. coli* and with *S. enterica serovar* Typhimurium. The tested yeast strains revealed no hemolytic activities and can therefore be considered safe. However, the probiotic potentials of *R. mucilaginosa* and *P. kudriavzevii* were lower than that of *S. cerevisiae*. So, taking into account these in vitro probiotic qualities, yeast strains isolated from *Rabilé* are promising strains and have the potential to be used as probiotic supplements. Further investigation will be performed on molecular aspects, functional attributes (sensibility of antibiotics, enzymatic profiling, and valuable metabolite profiling), and technological properties (preservation for extending the shelf life of final products). Additionally, our probiotic yeast strains with proven multifunctional properties could be useful in the development of functional foods which exhibit various health benefits.

## Figures and Tables

**Table 1:** Molecular identification of yeast isolates by RFLP analysis of the 5.8S-ITS regions.

Yeast Isolates	PCR ProductSize (bp)	Restriction Fragments (bp)			Identification
		*Hinf*I	*Hha*I	*Hae*III	
BB 1-2; BB 2-3; BB 3-2; BB 3-5; Bf 2-4; Bf 2-6; Bf 2-7; Dr 1-2; Dr 1-5; Dd 3-4; Fd 2-6; Fd 2-8; Ga 2-10; Ga 3-3	850	370 + 110	370 + 320 + 150	320 + 230 + 170 + 130	*Saccharomyces cerevisiae*
BB 3-7; Dr 1-8	510	220 + 150	210 +180 + 70	380 + 90	*Pichia kudriavzevii*
BB 3-3	620	340 + 210 + 70	300 + 220 + 90	410 + 210	*Rhodotorula mucilaginosa*

**Table 2:** Assessment of the capacities of yeast strains to grow at 37 °C and resist the simulated gastrointestinal conditions.

Strains	At 37 °C	Survival Rate (%)	
	DO600 nm	0.3% Pepsin and pH 2.5	0.3% Bile Salts
BB 3-7	2.58 ± 0.25 ^f^	96.96 ± 1.27 ^abc^	100.00 ± 0.00 ^a^
Dr 1-8	1.68 ± 0.11 ^g^	96.30 ± 1.42 ^abc^	100.00 ± 00 ^a^
BB 3-3	0.36 ± 0.01 ^h^	91.55 ± 4.97 ^cdef^	97.79 ± 1.07 ^b^
BB 1-2	2.73 ± 0.17 ^f^	99.98 ± 0.00 ^a^	98.65 ± 1.29 ^ab^
BB 2-3	3.32 ± 0.11 ^cd^	95.41 ± 6.97 ^abcde^	95.41 ± 2.74 ^c^
BB 3-2	3.30 ± 0.05 ^d^	88.46 ± 3.34 ^ef^	99.25 ± 0.65 ^ab^
BB 3-5	4.03 ± 0.10 ^ab^	91.22 ± 1.92 ^cdef^	99.36 ± 0.33 ^ab^
Bf 2-4	3.69 ± 0.16 ^bc^	86.01 ± 1.98 ^f^	100.00 ± 0.00 ^a^
Bf 2-6	2.87 ± 0.05 ^ef^	93.24 ± 5.41 ^abcde^	99.55 ± 0.56 ^ab^
Bf 2-7	2.90 ± 0.04 ^ef^	95.60 ± 3.41 ^abcd^	95.56 ± 0.43 ^c^
Dr 1-2	2.81 ± 0.12 ^f^	96.77 ± 1.94 ^abc^	98.73 ± 1.16 ^ab^
Dr 1-5	2.25 ± 0.00 ^f^	89.03 ± 9.45 ^def^	99.53 ± 0.80 ^ab^
Dd 3-4	3.19 ± 0.17 ^de^	92.36 ± 7.08 ^bcdef^	99.80 ± 0.33 ^ab^
Fd 2-6	2.68 ± 0.03 ^f^	97.82 ± 1.54 ^abc^	99.51 ± 0.60 ^ab^
Fd 2-8	3.36 ± 0.13 ^cd^	98.69 ± 1.37 ^ab^	100.00 ± 00 ^a^
Ga 2-10	4.27 ± 0.05 ^a^	96.59 ± 1.75 ^abc^	100.00 ± 00 ^a^
Ga 3-3	3.46 ± 0.08 ^cd^	96.95 ± 3.75 ^abc^	99.12 ± 1.51 ^ab^

Means and standard deviations (SDs) of three determinations. Values with the same letters are not significantly different at *p* = 0.05.

**Table 3:** Auto-aggregation (%) and co-aggregation with pathogens (%) and hydrophobicity (%) results for the yeast strains.

Strains	Auto-Aggregation (%)	Co-Aggregation (%)		Hydrophobicity (%)
		*E. coli*	*S. enterica serovar* Typhimurium	
BB 3-7	81.25 ± 1.83 ^ab^	63.27 ±3.60 ^abc^	74.06 ± 7.94 ^a^	60.97 ± 2.20 ^abc^
Dr 1-8	86.53 ± 4.82 ^a^	65.04 ± 7.56 ^abc^	67.44 ± 5.03 ^ab^	68.84 ± 5.23 ^a^
BB 3-3	89.40 ± 2.30 ^a^	80.68 ± 9.53 ^a^	70.83 ± 5.05 ^a^	68.99 ± 5.37 ^a^
BB 3-2	87.17 ± 2.86 ^a^	65.37 ± 4.35 ^abc^	69.38 ± 3.05 ^a^	51.99 ± 4.61 ^bcde^
BB 1-2	70.20 ± 10.53 ^b^	50.15 ± 4.16 ^bcd^	66.38 ± 5.50 ^abc^	43.17 ± 5.07 ^e^
BB 2-3	79.89 ± 7.08 ^ab^	56.16 ± 5.56 ^bcd^	57.9 ± 6.08 ^abcd^	63.09 ± 5.18 ^ab^
BB 3-5	85.43 ± 5.25 ^a^	36.06 ± 3.45 ^de^	45.71 ± 4.35 ^bcd^	59.17 ± 4.88 ^abcd^
Bf 2-4	84.38 ± 4.96 ^a^	24.92 ± 3.96 ^e^	40.89 ± 8.18 ^d^	60.11 ± 3.06 ^abc^
Bf 2-6	89.11 ± 2.40 ^a^	49.91 ± 4.58 ^bcd^	58.82 ± 6.70 ^abcd^	67.97 ± 2.12 ^a^
Bf 2-7	87.91 ± 3.47 ^a^	54.39 ± 4.90 ^bcd^	61.97 ± 2.71 ^abcd^	67.00 ± 2.45 ^a^
Dr 1-2	89.13 ± 3.55 ^a^	72.74 ± 8.02 ^ab^	68.76 ± 5.00 ^a^	60.06 ± 2.52 ^abcd^
Dr 1-5	81.48 ± 5.77 ^ab^	38.82 ± 10.8 ^de^	44.55 ± 7.09 ^cd^	64.53 ± 4.90 ^a^
Dd 3-4	87.55 ± 2.28 ^a^	66.33 ± 5.56 ^abc^	61.72 ± 5.19 ^abcd^	48.23 ± 0.94 ^de^
Fd 2-6	88.97 ± 1.54 ^a^	43.83 ± 7.03 ^cde^	59.74 ± 5.03 ^abcd^	70.73 ± 2.42 ^a^
Fd 2-8	88.93 ± 1.74 ^a^	45.43 ± 13.15 ^cde^	61.21 ± 9.99 ^abcd^	65.11 ± 4.25 ^a^
Ga 2-10	91.82 ± 1.96 ^a^	66.58 ± 7.02 ^abc^	72.78 ± 11.53 ^a^	63.04 ± 3.47 ^ab^
Ga 3-3	84.34 ± 2.27 ^a^	65.49 ±4.6 ^abc^	66.16 ± 5.29 ^abc^	49.89 ± 3.41 ^cde^

Means and standard deviations (SDs) of three determinations. Values with the same letters are not significantly different at *p* = 0.05.

**Table 4:** Antibacterial activities of the yeast strains (inhibitory zones in mm).

Strains	*E. coli* ATCC 8739	*S. aureus* ATCC 33592	*L. monocytogenes* ATCC 13932	*S. enteritidis* ATCC 13076	*S. typhimurium* ATCC 14028
Fd 2-6	23.66 ± 1.15 ^a^	24.33 ± 0.57 ^a^	26.66 ± 2.88 ^a^	27.00 ± 2.64 ^a^	24.66 ± 0.57 ^a^
Dd 3-4	23.33 ± 2.88 ^a^	21.33 ± 2.30 ^ab^	20.33 ± 0.57 ^bc^	26.00 ± 1.00 ^a^	25.00 ± 1.00 ^a^
Ga 2-10	21.66 ± 2.88 ^ab^	21.66 ± 0.57 ^ab^	20.00 ± 0.00 ^bc^	24.33 ± 1.15 ^ab^	20.33 ± 0.57 ^bc^
BB 3-2	20.00 ± 0.00 ^ab^	20.00 ± 0.00 ^abc^	30.00 ± 0.00 ^a^	25.00 ± 0.00 ^ab^	25.00 ± 0.00 ^a^
Bf 2-6	20.00 ± 0.00 ^ab^	20.00 ± 0.00 ^abc^	21.33 ± 1.15 ^b^	24.66 ± 0.57 ^ab^	21.33 ± 1.15 ^ab^
Fd 2-8	19.33 ± 1.15 ^abc^	20.00 ± 0.00 ^abc^	15.00 ± 0.00 ^de^	20.00 ± 0.00 ^cd^	16.33 ± 1.52 ^def^
Ga 3-3	19.33 ± 1.15 ^abc^	19.33 ± 0.57 ^bcd^	15.00 ± 0.00 ^de^	18.66 ± 1.15 ^cd^	16.33 ± 0.57 ^def^
Bf 2-4	17.33 ± 2.30 ^bcd^	20.00 ± 0.00 ^abc^	20.00 ± 0.00 ^bc^	21.33 ± 1.15 ^bc^	17.66 ± 2.08 ^bcde^
BB 1-2	15.00 ± 0.00 ^cd^	16.66 ± 2.88 ^cde^	17.33 ± 2.51 ^cd^	20.00 ± 2.00 ^cd^	17.33 ± 2.51 ^cde^
Dr 1-2	15.00 ± 0.00 ^cd^	16.66 ± 2.88 ^cde^	20.00 ± 0.00 ^bc^	17.33 ± 0.57 ^de^	17.66 ± 0.57 ^bcde^
BB 3-5	13.66 ± 1.52 ^de^	14.33 ± 0.57 ^ef^	16.00 ± 1.73 ^d^	16.33 ± 0.57 ^de^	14.33 ± 0.57 ^efg^
BB 2-3	13.33 ± 2.08 ^de^	14.33 ± 0.57 ^ef^	12.00 ± 1.73 ^e^	17.33 ± 1.15 ^de^	13.33 ± 1.15 ^fg^
Bf 2-7	13.00 ± 1.00 ^de^	15.00 ± 0.00 ^def^	16.66 ± 0.57 ^cd^	17.66 ± 2.51 ^cd^	18.33 ± 0.57 ^bcd^
Dr 1-5	10.00 ± 0.00 ^e^	11.00 ± 3.60 ^f^	14.33 ± 1.15 ^de^	13.66 ± 1.52 ^e^	10.66 ± 1.15 ^g^
BB 3-7	5.33 ± 0.57 ^f^	6.33 ± 0.57 ^g^	5.33 ± 0.57 ^f^	5.33 ± 0.57 ^f^	4.33 ± 1.52 ^h^
Dr 1-8	4.00 ± 1.73 ^fg^	5.00 ± 0.00 ^g^	6.00 ± 1.00 ^f^	5.00 ± 0.00 ^f^	4.00 ± 1.73 ^h^
BB 3-3	0.00 ± 0.00 ^g^	0.00 ± 0.00 ^h^	0.00 ± 0.00 ^g^	0.00 ± 0.00 ^g^	0.00 ± 0.00 ^i^

Means and standard deviations (SDs) of three determinations. Values with the same letters are not significantly different at *p* = 0.05.

## Data Availability

All data are available in this manuscript.
